# Disulfiram’s Anti-Inflammatory and Antibacterial Effects in Fixed-Dose Combination Therapy for Scabies

**DOI:** 10.3390/pharmaceutics18091172

**Published:** 2026-09-16

**Authors:** Carmen Salavastru, Angela Hernández-Martín, Sanja Bulut, Laura Fernàndez Fajardo, Aymée Robainas Barcia, Maria Lajarin-Reinares

**Affiliations:** 1 Pediatric Dermatology Department, Colentina Clinical Hospital, Carol Davila University of Medicine and Pharmacy, 050474 Bucharest, Romania; carmen.salavastru@umfcd.ro; 2 Department of Dermatology, Hospital Infantil Universitario Niño Jesús, 28009 Madrid, Spain; ahernandez@aedv.es; 3 Bioglan AB, Borrgatan 31, 21124 Malmö, Sweden; sanja.bulut@bioglan.se; 4 Faculty of Sciences and Biosciences, Autonomous University of Barcelona, 08193 Bellaterra, Spain; laura.fdez.fajardo@gmail.com; 5 Global Medical, Reig Jofre Laboratories, 08970 Sant Joan Despí, Spain; arobainas@reigjofre.com; 6 Translational Research, Reig Jofre Laboratories, 08970 Sant Joan Despí, Spain

**Keywords:** scabies, disulfiram, benzyl benzoate, antibacterial activity, keratinocyte inflammation

## Abstract

**Background/Objectives:** Scabies is a highly contagious parasitic skin infestation in which mite infestation, epidermal barrier disruption, secondary bacterial infection, and local inflammation collectively contribute to disease burden. This study investigated whether disulfiram, a component of a fixed-dose benzyl benzoate formulation, may provide local biological activity relevant to scabies-associated complications. **Methods:** Ex vivo permeation of the formulation was assessed in dermatomed porcine skin, with disulfiram retention in the stratum corneum and dermatomed skin measured against minimum inhibitory concentrations (MICs) for *Staphylococcus aureus* and *Streptococcus pyogenes*. Cytotoxicity of the formulation was evaluated in HEK001 keratinocytes across tested concentrations. In vitro antibacterial activity of disulfiram was tested against both Gram-positive species. Transcriptomic profiling was performed on keratinocytes exposed to the formulation. **Results:** Benzyl benzoate permeation was measurable in the ex vivo model, whereas disulfiram was not detected in the receptor phase under the experimental conditions. Disulfiram was retained in the stratum corneum and dermatomed skin at concentrations exceeding the MICs observed against *S. aureus* and *S. pyogenes*. The formulation was not cytotoxic in HEK001 keratinocytes at any tested concentration. Disulfiram showed in vitro antibacterial activity against both Gram-positive species. Transcriptomic profiling identified differential expression of genes involved in oxidative stress responses, detoxification, glutathione metabolism, innate immune signaling, and inflammatory mediator regulation. **Conclusions:** These findings support a mechanistic rationale for disulfiram as a locally retained component with potential therapeutic relevance to bacterial infection and keratinocyte inflammation in scabies.

## 1. Introduction

Scabies is a parasitic skin infestation caused by *Sarcoptes scabiei* var. *hominis*. It is a significant dermatological and public health concern, particularly in overcrowded and resource-limited settings [1,2]. The World Health Organization estimates that 200 million individuals are affected at any given time, accounting for over 400 million cases annually [3].

The clinical burden of scabies extends beyond parasite survival in the stratum corneum (SC). Pruritus, epidermal barrier disruption, eczematization and secondary bacterial infection are major contributors to morbidity and may persist after mite eradication [4,5]. Accordingly, the International Alliance for the Control of Scabies recognizes scabies as a heterogeneous inflammatory and pruritic disorder requiring a standardized clinical classification [1,4].

Secondary bacterial infection is frequently associated with *Staphylococcus aureus* (*S. aureus*) and *Streptococcus pyogenes* (*S. pyogenes*) [6], which can cause impetigo, cellulitis, abscess formation, acute post-streptococcal glomerulonephritis and rheumatic heart disease [7,8,9,10]. Mite-induced SC disruption facilitates pathogen penetration, while mite-derived complement inhibitors support bacterial persistence [11]. These interactions suggest that topical treatments should address not only mite eradication but also the inflammatory and microbiological consequences of infestation.

Topical permethrin, oral ivermectin and benzyl benzoate (BB) are considered first-line treatments for scabies [12,13]. Treatment selection may be influenced by age, pregnancy, disease severity, availability, tolerability and outbreak context [12,13,14]. Treatment failure and reduced susceptibility to permethrin and ivermectin have prompted interest in optimizing topical therapies [15]. Options are particularly limited in newborns and in pregnant or lactating women. In many countries, permethrin is not recommended in infants under 2 months, and ivermectin is generally restricted to children weighing at least 15 kg [16,17]. In this context, BB-based formulations have been used effectively in children younger than 2 years [18].

Tenutex^®^ (TNX) is a fixed-dose cutaneous emulsion that contains disulfiram (D; 20 mg/g) and BB (225 mg/g), indicated for scabies in adults and children in Nordic markets, including children under 2 years when the diagnosis is established by a physician [19,20]. D is a thiuram disulfide with recognized acaricidal activity, related to the sulfur-based scabicides long used topically and still recommended [7,12,21], and is also used orally as an aldehyde dehydrogenase inhibitor. It exhibits metal-chelating and thiol-reactive properties associated with antibacterial, redox- and inflammation-modulating effects [22,23].

Despite the long-standing clinical use of the fixed-dose D/BB combination, the contribution of D within the formulation remains poorly characterized. Three evidence gaps are relevant to its clinical positioning. First, the depth-resolved cutaneous distribution of D after topical application of the combination to human skin has not been established. Specifically, it remains unclear whether D is predominantly retained within the superficial skin compartments, where mites reside and secondary bacterial colonization may occur, or whether it penetrates deeper tissues and potentially reaches the systemic circulation. Second, although D has documented antibacterial activity against Gram-positive organisms in other contexts, its activity against the pathogens most frequently implicated in scabies-associated secondary infection has not been related to concentrations achievable in the skin after topical application of the combination. Third, the effect of the combination on keratinocyte inflammatory and oxidative-stress signaling, processes central to the persistent pruritus and eczematization that follow mite eradication, has not been examined.

Therefore, we hypothesized that, within the fixed-dose combination, D may provide therapeutic value beyond its antiparasitic function by exerting antibacterial activity against scabies-associated pathogens and modulating inflammatory and oxidative-stress responses. The specific objectives were: (i) to determine the permeation and cutaneous retention of D and BB in an ex vivo dermatomed porcine skin model, (ii) to assess the cytotoxicity of the fixed-dose combination in human keratinocytes, (iii) to determine the in vitro antibacterial activity of D against *S. aureus* and *S. pyogenes* and to relate the resulting MICs to the concentrations retained in skin, and (iv) to characterize the keratinocyte transcriptional response to the fixed-dose combination, with particular attention to oxidative stress and inflammatory pathways.

## 2. Materials and Methods

### 2.1. Materials

Formulations. Tenutex^®^ (TNX), the fixed-dose cutaneous emulsion containing disulfiram (D) 20 mg/g and benzyl benzoate (BB) 225 mg/g, was kindly provided by Bioglan AB (Malmö, Sweden; batch/lot number: L521A; expiration date: 02-2028). The qualitative composition of the excipient base is described in the Summary of Product Characteristics [20]. BB and D analytical standards were obtained from Sigma-Aldrich (Saint Louis, MO, USA).

Reagents. Phosphate-buffered saline (PBS), dimethyl sulfoxide (DMSO), gentamicin sulfate and clindamycin were purchased from Sigma-Aldrich (Saint Louis, MO, USA). Methanol and KH_2_PO_4_ were obtained from Scharlab, S.L. (Sentmenat, Spain). Hydroxypropyl-β-cyclodextrin (HPCD) was supplied by Pracofar, S.L. (Martorell, Spain). Ethanol, L-glutamine, human epidermal growth factor (hEGF), keratinocyte serum-free medium, and sodium dodecyl sulfate (SDS) were obtained from Thermo Fisher Scientific (Waltham, MA, USA). Mueller–Hinton broth (MHB) was obtained from Condalab (Madrid, Spain), and lysed horse blood from Thermo Fisher Scientific (Landsmeer, The Netherlands). All solvents and reagents used for the High-Performance Liquid Chromatography (HPLC) were of chromatographic grade.

Biological materials. Porcine abdominal skin was obtained from a local slaughterhouse (Barcelona, Spain) as a food-industry by-product. The HEK001 human epidermal keratinocyte line (CRL-2404) was purchased from the American Type Culture Collection (ATCC, Manassas, VA, USA). Reference strains *S. aureus* (ATCC^®^ 25923) and *S. pyogenes* (ATCC^®^ 12344) were supplied by ATCC through LGC Standards, S.L.U. (Barcelona, Spain).

Consumables and instrumentation. Vertical Franz diffusion cells were obtained from VidraFoc (Barcelona, Spain), and a CG1371 dermatome from Nouvag AG (Goldach, Switzerland) was used. Transepidermal water loss (TEWL) was measured using a VapoMeter device (model SWL5, Delfin Technologies, Kuopio, Finland). Tesa^®^ 4101 PV2 adhesive tape was obtained from Tesa (Shanghai, China), and a JP Selecta™ ultrasonic bath from JP Selecta (Abrera, Spain). A MagNA Lyser homogenizer was obtained from Roche (Sant Cugat del Vallès, Spain), and a Victor X3 luminometer from PerkinElmer (Waltham, MA, USA). The NanoDrop™ Lite spectrophotometer, PxE Thermal Cycler, and GeneChip^®^ Clariom™ S Human Array were obtained from Thermo Fisher Scientific (Waltham, MA, USA). The RNeasy^®^ Plus Mini Kit was obtained from Qiagen (Hilden, Germany). Chromatographic analyses were performed using a Waters 2695 Separations Module coupled to a Waters model 2996 PDA detector (Waters Corporation, Milford, MA, USA). CellTiter-Glo^®^ Luminescent Cell Viability Assay was supplied by Promega Corporation (Madison, WI, USA). Microarray data were acquired and processed with Affymetrix software version 4.0 (Affymetrix, Santa Clara, CA, USA).

### 2.2. Ex Vivo Skin Absorption Experiment with Porcine Skin

Porcine abdominal skin samples (*n* = 11) were dermatomed to a thickness of 0.5 mm. Ex vivo permeation studies were performed using vertical Franz diffusion cells with an effective diffusion area of 1.54 cm^2^ and a receptor compartment of 12 mL. Due to the low aqueous solubility of disulfiram, 15% HPCD at pH 5.5 was added to the receptor medium to improve drug solubilization and maintain sink conditions throughout the permeation study, while avoiding organic cosolvents that could alter skin-barrier properties. The same receptor medium has been used previously with this fixed-dose formulation [24]. The receptor medium was maintained at 32 °C and stirred at 500 rpm. At the end of each experiment, the receptor medium was recovered and its actual volume measured. This measured volume was used to correct the calculated permeated amounts. Skin integrity was confirmed by TEWL (<15 g/m^2^/h). A formulation dose of 5.6 mg (~3.65 mg/cm^2^) was applied to the donor compartment [25]. Samples (300 µL) were collected at 1, 2, 3, 4, 5, 20, 21, 22, 23, and 24 h. The sampling schedule was established based on in-house preliminary method-development experiments, which showed no relevant changes in the permeation rate between 5 and 20 h, and is consistent with OECD Test Guideline 428. This guideline specifies that the receptor-fluid sampling frequency should allow the absorption profile of the test substance to be presented graphically over a sampling period of normally 24 h [26]. Sampling was therefore concentrated in the initial and final phases to characterize the permeation profile while avoiding unnecessary disturbance of the receptor medium. The withdrawn volume was replaced with pre-warmed receptor medium.

Samples were then analyzed by HPLC as previously described [24]. A Waters 2695 Separations Module coupled to a Waters model 2996 PDA detector was used with a C18 (4.6 × 150 mm, 3 µm; Thermo Scientific, Barcelona, Spain) column maintained at 30 °C. The mobile phase consisted of KH_2_PO_4_ (1.7 mg/mL, pH 5.5) and methanol (30:70, *v*/*v*), and was delivered isocratically at 1.2 mL/min. The injection volume was 40 µL and detection was performed at 215 nm. The method was validated in accordance with the ICH Q2 guidelines [27]. The validated concentration ranges were 0.080–15.85 µg/mL for D and 0.0899–71.97 µg/mL for BB. Within these intervals, method performance was assessed in terms of linearity, intermediate precision and accuracy. The limit of quantification (LOQ) and the stability of the prepared solutions during storage in the HPLC autosampler were also evaluated.

### 2.3. In Vitro Cytotoxicity Evaluation

#### 2.3.1. Cell Culture and Maintenance

Human epidermal keratinocytes HEK001 (CRL-2404), an adult skin keratinocyte line immortalized with human papillomavirus type 16 E6/E7, were obtained from the ATCC. Cells were routinely maintained in keratinocyte serum-free medium (K-SFM) supplemented with 2 mM L-glutamine and 5 ng/mL hEGF, in accordance with the supplier’s recommendations for this cell line. Cultures were grown in 75 cm^2^ tissue-culture flasks at 37 °C in a humidified atmosphere of 95% air and 5% CO_2_, and the medium was renewed twice weekly.

Subculturing was performed when cultures reached 70–80% confluence, before the monolayer became fully confluent, to preserve the proliferative phenotype of the keratinocytes. Monolayers were rinsed with Ca^2+^- and Mg^2+^-free PBS and detached with 2–3 mL of 0.05% trypsin–0.53 mM EDTA for 5–15 min at 37 °C; flasks were not agitated during detachment in order to avoid cell clumping. Detached cells were collected by centrifugation at 200× *g* for 5 min, resuspended in fresh complete K-SFM and reseeded at a subcultivation ratio of 1:3 to 1:4. Cell number and viability were determined at each passage by trypan blue dye exclusion, and only cell suspensions with a viability above 90% were used for seeding.

#### 2.3.2. Cytotoxicity Assay

Cells were harvested as described above and seeded into flat-bottom, white-walled 96-well plates at 10,000 cells/well in 50 µL of complete K-SFM (*n* = 6 replicate wells per condition). The outermost wells of each plate were filled with sterile PBS and excluded from the analysis in order to limit edge-related evaporation effects over the assay period. Plates were incubated for 24 h at 37 °C and 5% CO_2_ to allow attachment and recovery before treatment.

After attachment, a 100 µL aliquot of medium containing D and BB was added to each well, yielding eight two-fold serial dilutions with final concentrations ranging from 33.36 to 1.95 µM for D and from 500 to 29.26 µM for BB, corresponding to the fixed 1:15 D/BB mass ratio of the formulation. The molar concentrations were calculated from the corresponding mass concentrations and dilution factors using molecular weights of 296.54 and 212.24 g/mol for D and BB, respectively.

Stock solutions were prepared in DMSO and diluted in culture medium so that the final DMSO concentration did not exceed 1% (*v*/*v*) in any well. Each plate included the following controls: untreated cells in complete medium (negative control, defined as 100% viability); cells exposed to the highest DMSO concentration used (vehicle control); cells exposed to 1% SDS (positive control, defined as 100% mortality); and cell-free wells containing medium alone and medium with each test concentration, used to correct for background luminescence and to verify that the test article did not optically interfere with the luminescent readout. Plates were then incubated for a further 72 h at 37 °C and 5% CO_2_ without medium renewal. Cells were exposed for 72 h to encompass approximately two to three HEK001 cell-doubling cycles and enable the detection of delayed or cumulative cytotoxic and antiproliferative effects that might not be apparent after shorter exposure periods.

Cell viability was assessed after 72 h using the CellTiter-Glo^®^ Luminescent Cell Viability Assay, which quantifies ATP as a surrogate marker of metabolically active cells. Plates and reagent were equilibrated to room temperature for approximately 30 min before use. A 100 µL volume of CellTiter-Glo^®^ reagent was added to each well; plates were mixed for 2 min on an orbital shaker to induce cell lysis and incubated for a further 10 min at room temperature, protected from light, to stabilize the luminescent signal. Luminescence was then recorded on a Victor X3 multilabel plate reader. Background luminescence obtained from the corresponding cell-free wells was subtracted from all readings, and the percentage of cell viability was calculated as follows:

Cell viability (%) = L_experimental value_ − L_positive control_/L_negative control_ − L_positive control_ × 100

where L_experimental value_ is the luminescence of the treated sample, L_negative control_ is the mean luminescence of untreated cells, and L_positive control_ is the luminescence with 1% SDS-treated cells.

### 2.4. Antibiotic Susceptibility Testing

Antibacterial activity was evaluated against *S. aureus* (ATCC^®^ 25923) and *S. pyogenes* (ATCC^®^ 12344). Minimum inhibitory concentrations (MICs) were determined by broth microdilution in 96-well microplates in accordance with the Clinical and Laboratory Standards Institute (CLSI) guidelines [28]. The MIC was defined as the lowest concentration of the test compound that inhibited visible bacterial growth. Overnight cultures were adjusted to 0.5 McFarland and diluted 1:100 in the corresponding medium: MHB for *S. aureus* and MHB supplemented with 5% lysed horse blood for *S. pyogenes*. The bacterial suspensions were then treated with serial dilutions of the tested compounds. Gentamicin and clindamycin served as reference controls for *S. aureus* and *S. pyogenes*, respectively.

D stock solutions were prepared in ethanol at 10 mg/mL and were tested at 64 to 0.125 µg/mL following previous studies [22]. The final ethanol concentration in all wells was kept below 0.5% to minimize interference with bacterial growth. Gentamicin and clindamycin were tested at 512 to 0.125 µg/mL. Negative controls were prepared using the corresponding media containing ethanol without D, whereas positive controls contained bacteria in medium without D. Plates were sealed and incubated at 37 °C for 18–24 h. MIC assays were performed independently in triplicate.

### 2.5. Microarrays and Data Analysis

HEK001 keratinocytes were seeded in 6-well plates at 250,000 cells/well in 2 mL of keratinocyte serum-free medium supplemented with L-glutamine (99:1) and incubated for 24 h at 37 °C with 5% CO_2_. Cells were treated for 24 h with the fixed-dose combination emulsion containing D (6.59 µM) and BB (99.77 µM). Treatments were performed in duplicate (*n* = 2), and control wells (*n* = 3) received medium without emulsion.

Total RNA was extracted using the RNeasy^®^ Plus Mini Kit. RNA concentration and purity were assessed by measuring the 260/280 nm absorbance ratio with a NanoDrop^TM^ Lite spectrophotometer. Complementary DNA was synthesized using a PxE Thermal Cycler. Transcriptomic profiling was performed using the GeneChip^®^ Clariom S Human Array. Data were acquired and analyzed with the Affymetrix software.

### 2.6. Quantification of Disulfiram in Skin After Permeation Experiments

#### 2.6.1. Tape-Stripping Study

After the permeation experiment, five of the eleven Franz cells were used to quantify D retained in the SC. Residual emulsion was removed with a PBS-soaked swab. After 24 h, adhesive tape strips were applied to the diffusion area under a constant pressure of 345 g for 10 s. Twenty consecutive strips were collected and pooled as follows: strip 1, strip 2, strips 3–7, strips 8–12, strips 13–17, and strips 18–20. Each pool was placed in a 50 mL Falcon tube containing 4 mL of mobile phase, sonicated for 15 min in an ultrasonic bath and analyzed by HPLC. Method selectivity was confirmed, with no interfering chromatographic peaks. SC recovery was determined gravimetrically by weighing the strips before and after extraction.

#### 2.6.2. Determination of the Concentration Retained in the Dermatomed Skin

After tape-stripping, the remaining dermatomed skin was used to determine the retained D. For each of the replicates (*n* = 5), 10 mg of treated skin was placed in a MagNa Lyser instrument with 600 µL of mobile phase and homogenized for five cycles of 90 s at 6500 rpm. The homogenates were then analyzed by HPLC.

### 2.7. Statistical Analysis

Quantitative data are expressed as mean ± standard deviation (SD). The number of replicates (*n*) for each experiment corresponds to independent biological replicates and is indicated in the respective section and figure legend: *n* = 11 skin samples for ex vivo permeation, *n* = 6 wells per concentration for the cytotoxicity assay, three independent assays for MIC determination, and *n* = 2 treated wells versus *n* = 3 control wells for transcriptomic profiling.

Permeation parameters were derived from the cumulative amount of BB permeated per unit area plotted against time. The steady-state flux (J) was calculated as the slope of the linear portion of the curve by least-squares linear regression, and the lag time (Tlag) as the intercept of the regression line on the time axis. The coefficient of determination (R^2^) was used to assess linearity of the permeation profile. The permeability coefficient (Kp), permeation constant (P) and diffusion coefficient (Dif) were calculated from J and the applied donor concentration according to Fick’s first law, and are reported as mean ± SD.

Cell viability was expressed as a percentage of the untreated control after normalization to the 1% SDS positive control. Results are presented as mean ± SD of six replicates per concentration. Variability between replicates is reported as SD.

MIC values were determined by broth microdilution in three independent assays performed on separate days and are reported as the concentration reproducibly inhibiting visible growth across replicates. As MIC is a discrete, non-continuous endpoint, no inferential statistical testing was applied.

For transcriptomic analysis, differential gene expression between treated and control keratinocytes was assessed using an unpaired two-tailed Student’s *t*-test. Genes were considered differentially expressed when the absolute fold change was >1.5 and the nominal *p*-value was ≤0.05. Given the exploratory nature of the microarray experiment and the limited number of replicates, *p*-values were not adjusted for multiple comparisons. Accordingly, transcriptomic findings are reported as hypothesis-generating and were interpreted at the level of functionally coherent gene sets rather than individual transcripts. All statistical analyses were performed using Minitab 7 statistical software (Minitab, Inc., State College, PA, USA).

## 3. Results and Discussion

### 3.1. Ex Vivo Skin Absorption Experiment with Porcine Skin

**Figure 1:**
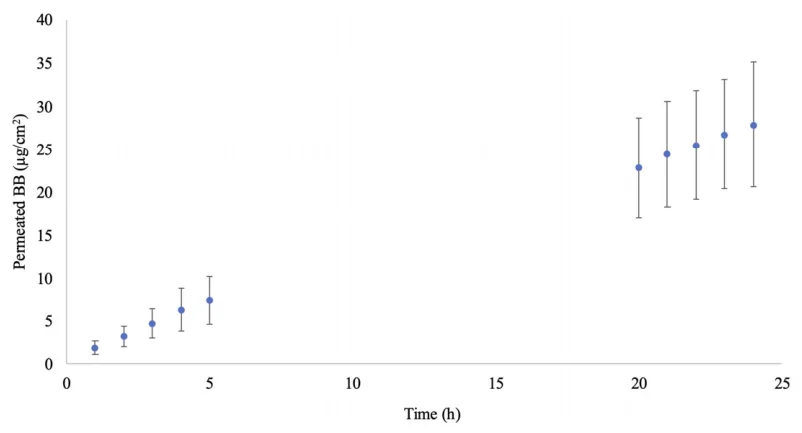
Ex vivo permeation of BB in dermatomed porcine skin. Scatter plot showing cumulative BB permeation in dermatomed porcine skin over 24 h after administration of 3.65 mg/cm^2^ emulsion (*n* = 11). Points represent mean permeated BB amounts at each sampling time, with error bars indicating SD. Abbreviations: BB, benzyl benzoate; µg, microgram; cm, centimeter; h, hour; SD, standard deviation; mg, milligram; cm, centimeter.

BB was detected in receptor-phase samples at all time points, whereas D was not detected in any chromatographic replicate (Figure 1).

BB showed measurable permeation across dermatomed porcine skin, with a steady-state flux of 1.34 ± 0.43 µg/cm^2^/h, a low lag time of 0.43 ± 0.12 h and a permeability coefficient of 5.57 × 10^−6^ ± 2.02 × 10^−6^ cm/h (Table 1). The high linearity of the cumulative permeation curve (R^2^ = 0.993) was consistent with a diffusion-driven profile.

These results are consistent with human skin data previously reported for the same D/BB formulation [24]. Detection of BB in the receptor phase is consistent with its role as the permeating antiparasitic component. Its physicochemical properties, including low molecular weight (212.24 Da), moderate lipophilicity (log *p* value 3.9), low melting point (21 °C) and limited hydrogen-bonding capacity, also favor permeability [29]. The high diffusion coefficient (Table 1) suggests diffusion-driven permeation with a small partitioning contribution. Recent clinical studies confirm the high efficacy of BB in both adults and children, even in cases of reduced permethrin susceptibility [30,31]. The fixed-dose D/BB emulsion has shown clinical efficacy in randomized trials, with cure rates influenced by its single whole-body application, whereas comparators were administered more frequently [32,33,34]. This is consistent with the TNX Summary of Product Characteristics, where a single application is sufficient in most cases, with a second reserved for severe cases [20].

D was not detected despite having physicochemical descriptors that could support permeation, likely reflecting its low formulation concentration (2.0%), SC retention and gradual release from the matrix [35,36]. The lack of measurable permeation may be advantageous, since systemic exposure to D has been associated with adverse effects after oral administration [19,33,37,38].

### 3.2. In Vitro Cytotoxicity Evaluation

**Figure 2:**
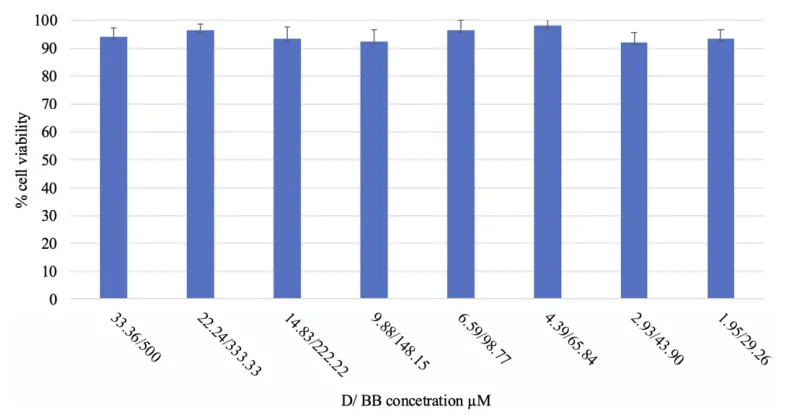
HEK001 cell viability after exposure to D/BB combination. Vertical bar chart showing HEK001 cell viability after 72 h of exposure to D/BB combination (*n* = 6). Bars represent mean cell viability expressed as percentage of control, with error bars indicating SD. **Abbreviations:** D, disulfiram; BB, benzyl benzoate; µM, micromolar; SD, standard deviation.

No cytotoxic effects were observed for the fixed-dose D/BB combination at any concentration (Figure 2).

Cell viability exceeded 90% throughout, with low variability between replicates (SD < 5.00%), indicating good tolerability under the conditions tested, which is relevant because scabies formulations are commonly applied to inflamed, excoriated or barrier-compromised skin. Previous data similarly showed that BB did not decrease cell viability across concentrations [24]. This is relevant since BB is the most concentrated component of the formulation and shows measurable skin permeation [24,39].

D is the differentiating active ingredient, included for its antiparasitic activity and potential antibacterial, redox-modulating and inflammation-related properties [23,40,41]. TNX has been used in Scandinavia for years, with published data supporting local tolerance at the prescribed therapeutic dose [19,33,37]. The lack of cytotoxicity is consistent with the formulation’s known tolerability, although whether it translates into improved tolerability versus other scabicides requires controlled clinical comparisons.

### 3.3. Antibiotic Susceptibility Testing

D showed antibacterial activity against both strains, supporting its potential relevance to scabies-associated bacterial infection (Table 2).

*S. aureus* was the more susceptible species (MIC 8 µg/mL), whereas the MIC against *S. pyogenes* was 32 µg/mL. These values are consistent with previously published data for both species [22,42,43].

No clinical susceptibility breakpoints have been established for D [28]. Thus, MIC values cannot be interpreted as susceptible, intermediate or resistant categories. Nevertheless, the observed activity is consistent with previous studies [22,40,42,43], suggesting that D may provide a local antibacterial effect relevant to the secondary bacterial complications of scabies.

The activity against Gram-positive bacteria may involve complementary mechanisms such as Cu^2+^ chelation and interaction with cysteine thiol groups [23,44]. Cysteine residues are crucial for bacterial protein structure and function, including binding, enzymatic catalysis and disulfide bond formation [45,46]. Because Gram-positive bacteria lack an outer membrane and a protected periplasmic compartment, their surface and cell-envelope-associated proteins may be accessible to thiol-reactive compounds [47], accounting for the susceptibility of both species. D-mediated modification of cysteine residues may impair protein maturation or function and reduce bacterial viability.

Mass drug administration for scabies has been associated with a reduction in hospitalizations for skin and soft-tissue infections and in related primary-care presentations [48]. Community treatment of scabies substantially lowers the prevalence of impetigo caused by *S. aureus* and *S. pyogenes* [49]. However, since no trial has yet evaluated topical D for cutaneous bacterial infection, its contribution to clinical outcomes remains to be established.

### 3.4. Analysis of Differential Gene Expression Related to Inflammation

Transcriptomic analysis identified 117 differentially expressed genes in TNX-treated versus untreated keratinocytes (63 upregulated, 54 downregulated). Table 3 and Table 4 summarize those most relevant to antioxidant defense, detoxification, innate immune signaling and inflammatory mediator regulation.

AKR1C1 and AKR1C2 were the most strongly induced genes (fold changes of 2.26 and 2.20), suggesting activation of a keratinocyte detoxification program against reactive carbonyl and oxidative stress-associated metabolites. Both are induced in keratinocytes by UVB exposure and ROS [50,51]. AKR1C3 has also been linked to ROS metabolism and reactive carbonyl detoxification, reinforcing the role of AKR1C-mediated redox regulation in cutaneous inflammation, oxidative stress and tissue repair [52].

Upregulation of SLC7A11 (1.75-fold) and GCLC (1.58-fold) further supports a glutathione cytoprotective response. SLC7A11 mediates cystine uptake for glutathione biosynthesis [53], and GCLC encodes the catalytic subunit of the rate-limiting enzyme in glutathione synthesis [54]. CHAC1, encoding a γ-glutamylcyclotransferase that preferentially degrades reduced glutathione, was upregulated (1.85-fold), contributing to intracellular glutathione turnover and redox balance [55]. Together, these changes suggest enhanced cystine import, glutathione biosynthesis and glutathione cycling, consistent with keratinocyte survival, epidermal repair and Nrf2 antioxidant responses [56,57].

Other antioxidant and detoxification genes were induced, including CAT (1.63-fold), EPHX1 (1.52-fold) and NQO1 (1.54-fold). CAT detoxifies hydrogen peroxide and EPHX1 hydrolyzes reactive epoxide intermediates [58,59]. NQO1 reduces quinones to hydroquinones, limiting semiquinone formation and oxidative damage from redox cycling [60]. This response is consistent with Nrf2-mediated regulation of antioxidant defense, xenobiotic metabolism, redox balance and inflammatory control [61]. The induction of NQO1, a downstream marker of Nrf2 activation, is particularly relevant [60].

TNX suppressed classical pro-inflammatory mediators, including PTGS2 (0.64-fold), IL-24 (0.57-fold), IL-33 (0.65-fold) and CXCL5 (0.67-fold). PTGS2 encodes cyclooxygenase-2, key to prostaglandin biosynthesis [62], while IL24/IL33 encode cytokines associated with epithelial stress, cutaneous inflammation and barrier-associated immune responses [63,64], and CXCL5 is a neutrophil-recruiting chemokine [65]. In addition, IL7R (0.66-fold), involved in adaptive immune signaling [66], and ADAMTS1 (0.65-fold), involved in extracellular matrix remodeling [67], were also downregulated. The most strongly suppressed gene was AOX1 (0.55-fold), encoding aldehyde oxidase, which contributes to endogenous ROS production during substrate oxidation [68,69]. These changes support a selective shift towards reduced inflammatory amplification, neutrophil recruitment, tissue remodeling and ROS generation.

TNX also increased the expression of the pattern recognition receptor TLR6 (1.69-fold) and the negative modulator of IL-1 signaling, IRAK1BP1 (1.62-fold), indicating activation of innate immune sensing with concurrent regulatory control [70]. CXCL10, which recruits T lymphocytes and NK cells via CXCR3, was upregulated (1.51-fold), while CXCL5 was suppressed, suggesting a selective reprogramming of immune cell recruitment rather than broad inhibition of chemokine signaling [71].

TNFSF10, encoding TRAIL, was induced (1.55-fold). Beyond apoptosis, TRAIL mediates the clearance of activated neutrophils and pro-inflammatory macrophages [72], a signal compatible with inflammatory resolution in scabies. However, whether it enhances immune-cell clearance or accelerates recovery after mite eradication remains to be determined. Finally, REV-ERBα, encoded by NR1D1, adds complexity to the inflammatory profile [23]. Its decreased expression (0.63-fold) could relieve transcriptional repression of selected inflammatory targets.

Previous studies have shown that topical lindane increased serum malondialdehyde and reduced glutathione relative to permethrin [73], with antioxidant supplementation attenuating these alterations [74]. The upregulation of glutathione- and Nrf2-associated antioxidant defenses and downregulation of proinflammatory mediators present a mechanistically favorable profile. However, additional studies are required to confirm whether these transcriptional changes translate into clinical benefit. Overall, TNX produced a transcriptional profile consistent with antioxidant defense, glutathione turnover, detoxification and selective modulation of inflammatory and ROS-generating mediators, in line with the known redox-modulating properties of D.

### 3.5. Quantification of Disulfiram in Skin

Despite the absence of measurable D permeation, superficial penetration into the SC and epidermis cannot be excluded. Because mites localize mainly in the SC and the epidermal surface is relevant to bacterial contamination and inflammation, D retention was quantified in these compartments.

#### 3.5.1. Quantification of Disulfiram in SC Using the Tape-Stripping Technique

**Figure 3:**
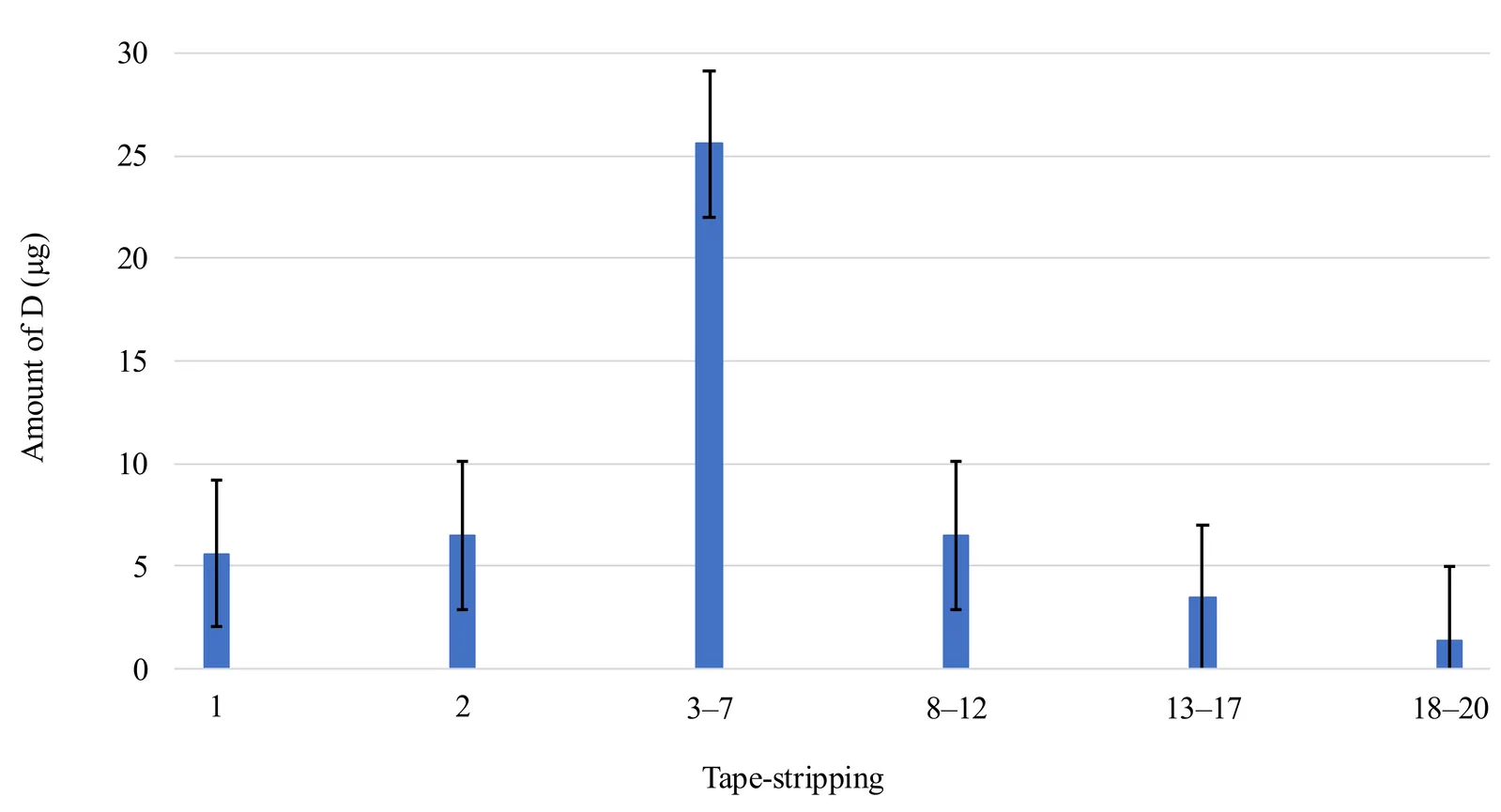
D retention in the stratum corneum after tape stripping. Vertical bar chart showing the amount of D recovered from sequential stratum corneum tape-strip groups after topical exposure (*n* = 5). Bars represent the mean amount of D recovered per strip group, with error bars indicating SD. Abbreviations: D, disulfiram; µg, microgram.

A total of 50 µg of D was recovered from the SC, representing 45% of the applied dose (110 µg) (Figure 3).

The mean recovered tissue mass was 2.5 ± 0.18 mg, yielding an estimated D concentration of 19 mg/mL. This exceeded the MIC values determined for *S. aureus* (8 µg/mL) and *S. pyogenes* (32 µg/mL) by 2375-fold and 594-fold, respectively. It was also above the 6.59 µM concentration used in the transcriptomic experiment, at which D modulated antioxidant and inflammatory gene expression in keratinocytes. These concentrations fall within the low-micromolar range in which anti-inflammatory effects of D have been reported in human cells and skin, including inhibition of inflammasome activation and IL-1β release [75,76,77]. Since scabies-associated lesions combine mite infestation with a marked inflammatory component, the ability to reach such concentrations in the skin supports the potential relevance of D in this setting. Thus, D was retained in the superficial compartment most relevant to mite localization and early bacterial contamination, where it may contribute to local antibacterial and inflammation-modulating activity.

#### 3.5.2. Disulfiram Concentration Retained in the Dermatomed Skin

A mean of 3.4 ± 1.4 µg of D was recovered from dermatomed skin, corresponding to 1.3 mg/mL. This concentration also exceeded the MICs for both organisms and the 6.59 µM concentration, further supporting the relevance of D in scabies-associated skin infection and inflammation.

Overall, D was largely retained within the superficial skin compartments, while BB permeated to a measurable extent, providing a mechanistic rationale for the fixed-dose formulation in which BB acts as the established antiparasitic component and D may add antibacterial and inflammation-modulating effects. However, direct evidence that retained D reduces bacterial colonization or local inflammation in vivo is not yet available and should be addressed in the future.

### 3.6. Study Limitations and Clinical Perspective

The present study is preclinical and mechanistic, relying on ex vivo and in vitro assays and therefore describes potential local biological activity rather than clinically validated outcomes. Dermatomed porcine skin is a well-established model of human barrier function but does not fully replicate human percutaneous absorption. Transcriptomic analyses were performed on a single immortalized keratinocyte cell line, reflecting associative gene expression changes without protein-level confirmation. Antibacterial evaluation was limited to reference strains under standardized conditions, which may limit extrapolation of the findings to clinically relevant settings. Clinical parameters such as secondary bacterial infection, skin inflammation, pruritus and treatment response were not assessed. Consequently, these findings should be interpreted as mechanistic evidence requiring further clinical validation. Future studies should determine whether these results translate into clinically meaningful benefits, such as reduced secondary infection, improved inflammation control, decreased pruritus and improved post-treatment recovery, and clarify the role of this combination in vulnerable populations with limited therapeutic alternatives.

## 4. Conclusions

In summary, under the ex vivo and in vitro conditions described in this study, D was not detected in the receptor phase within this fixed-dose formulation, yet it was retained in the SC and epidermis at concentrations exceeding those associated with antibacterial activity against *S. aureus* and *S. pyogenes*. The transcriptomic profile revealed a coordinated regulation of genes involved in oxidative stress response, glutathione metabolism, xenobiotic detoxification and inflammatory signaling, including the selective downregulation of key pro-inflammatory mediators. These transcriptional changes are consistent with an antioxidant and anti-inflammatory phenotype, although functional confirmation is required. By targeting secondary bacterial infection and cutaneous inflammation, D may broaden the mechanistic scope and clinical utility of the combination beyond its antiparasitic role in scabies management.

## Figures and Tables

**Table 1:** Permeation parameters of BB in porcine skin.

	Mean	SD
J (µg/cm^2^h)	1.3416	0.4255
R^2^	0.9930	0.0092
Kp (cm/h)	5.57 × 10^−6^	2.02 × 10^−6^
Tlag (h)	0.4328	0.1196
P (cm/h^2^)	1.53 × 10^−5^	3.81 × 10^−6^
Dif (1/h)	0.7571	0.0399

Quantitative variables are presented as mean and SD. Abbreviations: J, steady-state flux; µg, microgram; cm, centimeter; h, hour; R^2^, coefficient of determination; Kp, permeability coefficient; Tlag, lag time; P, permeation constant; Dif, diffusion coefficient; SD, standard deviation.

**Table 2:** Antibacterial activity of D compared with gentamicin and clindamycin.

Species	MIC					
	Disulfiram		Clindamycin		Gentamicin	
	(µg/mL)	(µM)	(µg/mL)	(µM)	(µg/mL)	(µM)
*S. pyogenes*	32	108	0.125	1	–	–
*S. aureus*	8	27	–	–	0.125	0.262

Abbreviations: MIC, minimum inhibitory concentration; µM, micromolar; µg, microgram; mL, milliliter.

**Table 3:** Upregulated genes related to antioxidant defense and immune signaling.

Gene Symbol	(TNX) Raw	(Control) Raw	Ratio	Upregulation
AKR1C1	342.08	156.58	2.26	126%
AKR1C2	367.94	172.22	2.20	120%
CHAC1	468.36	251.96	1.85	85%
SLC7A11	1294.21	727.61	1.75	75%
TLR6	105.41	59.77	1.69	69%
CAT	348.92	232.31	1.63	63%
IRAK1BP1	181.18	112.67	1.62	62%
GCLC	798.51	504.34	1.58	58%
TNFSF10	553.44	360.30	1.55	55%
NQO1	1780.95	1174.10	1.54	54%
EPHX1	387.87	235.71	1.52	52%
CXCL10	193.36	128.57	1.51	51%

Abbreviations: AKR1C1, aldo-keto reductase family 1 member C1; AKR1C2, aldo-keto reductase family 1 member C2; CHAC1, ChaC glutathione-specific gamma-glutamylcyclotransferase 1; SLC7A11, solute carrier family 7 member 11; TLR6, Toll-like receptor 6; CAT, catalase; IRAK1BP1, interleukin 1 receptor associated kinase 1 binding protein 1; GCLC, glutamate-cysteine ligase catalytic subunit; TNFSF10, TNF superfamily member 10; NQO1, NAD(P)H quinone dehydrogenase 1; EPHX1, epoxide hydrolase 1; CXCL10, C-X-C motif chemokine ligand 10; TNX, Tenutex.

**Table 4:** Downregulated genes related to inflammatory mediators and reactive oxygen species (ROS) production.

Gene Symbol	(TNX) Raw	(Control) Raw	Ratio	Upregulation
AOX1	110.07	198.98	0.55	−45%
IL24	324.68	571.80	0.57	−43%
NR1D1	289.14	464.75	0.63	−37%
PTGS2	984.22	1487.66	0.64	−36%
ADAMTS1	245.40	356.94	0.65	−35%
IL33	31.32	47.86	0.65	−35%
IL7R	661.60	977.41	0.66	−34%
CXCL5	1358.63	2006.04	0.67	−33%

Abbreviations: AOX1, aldehyde oxidase 1; IL24, interleukin 24; NR1D1, nuclear receptor subfamily 1 group D member 1; PTGS2, prostaglandin-endoperoxide synthase 2; ADAMTS1, ADAM metallopeptidase with thrombospondin motif 1; IL33, interleukin 33; IL7R, interleukin 7 receptor; CXCL5, C-X-C motif chemokine ligand 5; TNX, Tenutex.

## Data Availability

The data supporting the findings of this study are available from the corresponding author upon reasonable request. Public deposition is precluded because the data are related to a commercial product and are subject to intellectual property and confidentiality restrictions. The raw data or a minimal dataset can be provided for internal editorial evaluation upon request.

## Abbreviations

The following abbreviations are used in this manuscript:

ADAMTS1	ADAM metallopeptidase with thrombospondin motif 1
AKR1C1	Aldo-keto reductase family 1 member C1
AKR1C2	Aldo-keto reductase family 1 member C2
AKR1C3	Aldo-keto reductase family 1 member C3
AOX1	Aldehyde oxidase 1
ATCC	American Type Culture Collection
BB	Benzyl benzoate
CAT	Catalase
CHAC1	ChaC glutathione-specific gamma-glutamylcyclotransferase 1
cm	Centimeter
CO_2_	Carbon dioxide
COX-2	Cyclooxygenase-2
CXCL5	C-X-C motif chemokine ligand 5
CXCL10	C-X-C motif chemokine ligand 10
CXCR3	C-X-C chemokine receptor type 3
°C	Degree Celsius
D	Disulfiram
Dif	Diffusion coefficient
DMSO	Dimethyl sulfoxide
EPHX1	Epoxide hydrolase 1
GCLC	Glutamate-cysteine ligase catalytic subunit
h	Hour
HEK001	Human epidermal keratinocyte cell line
hEGF	Human epidermal growth factor
HPCD	Hydroxypropyl-β-cyclodextrin
HPLC	High-performance liquid chromatography
IL-24	Interleukin 24
IL-33	Interleukin 33
IL7R	Interleukin 7 receptor
IRAK1BP1	Interleukin 1 receptor associated kinase 1 binding protein 1
J	Steady-state flux
K-SFM	Keratinocyte serum-free medium
kg	Kilogram
Kp	Permeability coefficient
MHB	Mueller–Hinton broth
MIC	Minimum inhibitory concentration
mg	Milligram
mL	Milliliter
mm	Millimeter
NK	Natural killer (cells)
nm	Nanometer
NQO1	NAD(P)H quinone dehydrogenase 1
NR1D1	Nuclear receptor subfamily 1 group D member 1
Nrf2	Nuclear factor erythroid 2–related factor 2
P	Permeation constant
PBS	Phosphate-buffered saline
PTGS2	Prostaglandin-endoperoxide synthase 2
R^2^	Coefficient of determination
REV-ERBα	Reverse erythroblastosis virus receptor alpha
ROS	Reactive oxygen species
rpm	Revolutions per minute
*S. aureus*	*Staphylococcus aureus*
*S. pyogenes*	*Streptococcus pyogenes*
SC	Stratum corneum
SD	Standard deviation
SDS	Sodium dodecyl sulfate
SLC7A11	Solute carrier family 7-member 11
TEWL	Transepidermal water loss
Tlag	Lag time
TLR6	Toll-like receptor 6
TNFSF10	TNF superfamily member 10
TNX	Tenutex
TRAIL	TNF-related apoptosis-inducing ligand
µg	Microgram
µM	Micromolar
